# Identification and Characterization of Immunogene-Related Alternative Splicing Patterns and Tumor Microenvironment Infiltration Patterns in Breast Cancer

**DOI:** 10.3390/cancers14030595

**Published:** 2022-01-25

**Authors:** Shuang Guo, Xinyue Wang, Hanxiao Zhou, Yue Gao, Peng Wang, Hui Zhi, Yue Sun, Yangyang Hao, Jing Gan, Yakun Zhang, Jie Sun, Wen Zheng, Xiaoxi Zhao, Yun Xiao, Shangwei Ning

**Affiliations:** College of Bioinformatics Science and Technology, Harbin Medical University, Harbin 150081, China; guoshuang@hrbmu.edu.cn (S.G.); 2020020455@hrbmu.edu.cn (X.W.); zhouhanxiao@hrbmu.edu.cn (H.Z.); 102530@hrbmu.edu.cn (Y.G.); wpgqy@hrbmu.edu.cn (P.W.); zhihui@ems.hrbmu.edu.cn (H.Z.); 2019020354@hrbmu.edu.cn (Y.S.); 2019020369@hrbmu.edu.cn (Y.H.); 2019020361@hrbmu.edu.cn (J.G.); 2019020355@hrbmu.edu.cn (Y.Z.); 2019020387@hrbmu.edu.cn (J.S.); 2020020499@hrbmu.edu.cn (W.Z.); 2020020483@hrbmu.edu.cn (X.Z.)

**Keywords:** immunogene-related alternative splicing, tumor microenvironment, prognosis signatures, immuno/chemotherapy, breast cancer

## Abstract

**Simple Summary:**

Aberrant immunogene-related alternative splicing (IGAS) pattern plays a pivotal role in pathogenesis, progression, and tumor microenvironment. However, the IGAS pattern of post-transcriptional mechanisms in breast cancer remains limited. Here, we performed a systematic analysis of IGAS patterns in breast cancer to assess the association between aberrant IGAS events, prognosis signatures, AS regulatory network, immune cell infiltration level and its marker gene expression, sensitivity to immunotherapy and chemotherapy, and heterogeneity of IGAS clusters. Generally, we demonstrated the prognostic signatures for IGAS events and immune cells, which were valuable information for breast cancer patients in predicting survival and directing immunotherapy and chemotherapy.

**Abstract:**

Alternative splicing (AS) plays a crucial role in tumor development and tumor microenvironment (TME) formation. However, our current knowledge about AS, especially immunogene-related alternative splicing (IGAS) patterns in cancers, remains limited. Herein, we identified and characterized post-transcriptional mechanisms of breast cancer based on IGAS, TME, prognosis, and immuno/chemotherapy. We screened the differentially spliced IGAS events and constructed the IGAS prognostic model (*p*-values < 0.001, AUC = 0.939), which could be used as an independent prognostic factor. Besides, the AS regulatory network suggested a complex cooperative or competitive relationship between splicing factors and IGAS events, which explained the diversity of splice isoforms. In addition, more than half of the immune cells displayed varying degrees of infiltration in the IGAS risk groups, and the prognostic characteristics of IGAS demonstrated a remarkable and consistent trend correlation with the infiltration levels of immune cell types. The IGAS risk groups showed substantial differences in the sensitivity of immunotherapy and chemotherapy. Finally, IGAS clusters defined by unsupervised cluster analysis had distinct prognostic patterns, suggesting an essential heterogeneity of IGAS events. Significant differences in immune infiltration and unique prognostic capacity of immune cells were also detected in each IGAS cluster. In conclusion, our comprehensive analysis remarkably enhanced the understanding of IGAS patterns and TME in breast cancer, which may help clarify the underlying mechanisms of IGAS in neoplasia and provide clues to molecular mechanisms of oncogenesis and progression.

## 1. Introduction

Breast cancer is the most common malignancy among women and is a heterogeneous disease at the molecular level [1]. Breast cancer treatment is multidisciplinary, since therapeutic strategies depend on different molecular subtypes [2]. Emerging immunotherapies had shown promise in many solid tumors, including melanoma [3] and non-small cell lung cancer [4], and an important role in breast cancer is evolving. However, improving the overall survival of breast cancer patients remains a common challenge worldwide.

The tumor microenvironment (TME) is a highly heterogeneous and dynamic network, which influences tumor initiation and progression by promoting cancer cell survival, migration, metastasis, chemoresistance, and evading immune responses [5]. An increasing number of studies had suggested that immune cell subtypes have demonstrated differential prognostic values. For example, there was a significant correlation between the high occupancy of T-cells and a good clinical outcome in breast cancer [6]. Nevertheless, the prognostic values of multiple immune cells remain unclear.

Alternative splicing (AS) is a widespread regulatory mechanism of gene expression in eukaryotic transcripts that significantly contributes to the diversity of gene and protein functions by removing introns and ligating exons to form pre-mRNA [7]. Approximately 90–95% of human protein-coding genes in genome-wide studies had undergone AS [8]. The primary mechanism of AS in cancer has been firmly established in recent extensive genomic and functional studies, including immune escape, metastasis, cell proliferation, apoptosis, hypoxia, and angiogenesis [9]. However, AS events, especially those associated with immunogenes, have not been sufficiently studied in breast cancer.

Over the past decades, studies have paid attention to understanding the crucial role of transcriptional regulation in the immune system. However, less attention has been paid to the post-transcriptional mechanisms of gene regulation as important regulators of immune cell function. In particular, immune cells are constantly dividing and differentiating throughout their lifetime, and they particularly need to utilize AS to ensure transcriptional diversity and regulation of gene expression [10]. Therefore, we urgently need to identify immunogene-related alternative splicing (IGAS) events to predict patient prognostic ability and treatment response and to understand the regulatory role of AS in the immune system.

In this study, we systematically profiled the IGAS events and the TME in breast cancer from TCGA. We implemented a series of rigorous screens to identify IGAS events and explored the relationship of IGAS events to clinical outcomes and immune characteristics. The low-risk group had better prognostic outcomes, higher levels of immune infiltration, and higher sensitivity to immunotherapy and chemotherapy than the high-risk group, potentially assisting oncologists in clinical decision-making. In addition, AS regulatory networks with complex cooperative or competitive relationships between splicing factors and AS events provided an insight into the diversity of AS. Notably, we distinguished highly heterogeneous IGAS clusters based on IGAS events, which were significantly associated with overall survival status, immune characteristics, and tumor-infiltrating immune cells. This study will help identify reliable prognostic signatures and develop appropriate therapeutic approaches to breast cancer.

## 2. Material and Methods

### 2.1. Data Acquisition and Processing

TCGA SpliceSeq (https://bioinformatics.mdanderson.org/TCGASpliceSeq/, accessed on 26 August 2020) provides a comprehensive and detailed view of mRNA AS patterns [11]. There are seven types of AS events, including Alternate Acceptor site (AA), Alternate Donor site (AD), Alternate Promoter (AP), Alternate Terminator (AT), Exon Skip (ES), Mutually Exclusive Exons (ME), and Retained Intron (RI). The Percent Spliced In (PSI) value is a common and intuitive ratio to quantify splicing events, representing the number of reads present for transcriptional elements divided by the total number of reads covering splicing events. We obtained the AS patterns of protein-coding genes in breast cancer samples according to the percentage of samples with PSI value ≥ 75% and a minimum PSI standard deviation ≥ 0.10.

RNA-Seq data and corresponding clinicopathological characteristics (including T, N, M, pathological stage, number of positive lymph nodes, hormonal receptor status, overall survival (OS), and relapse-free survival (RFS)) of breast cancer were obtained from The Cancer Genome Atlas (TCGA, https://tcga-data.nci.nih.gov/tcga/, accessed on 26 August 2020).

The ESTIMATE (https://bioinformatics.mdanderson.org/estimate/, accessed on 26 August 2020) algorithm predicts tumor purity and the presence of infiltrating immune/stromal cells in tumor tissue based on single-sample genomic enrichment analysis (ssGSEA) and generates immune and stromal scores [12]. We employed the ESTIMATE algorithm to calculate the immune score and stromal score for each breast cancer sample.

Then, samples were included due to the inclusion criteria: (i) OS time of patient >30 days; (ii) primary tumor samples; (iii) samples included in all the above three databases. Finally, 918 primary breast cancer samples and 113 corresponding normal samples with 11,307 AS events were enrolled for subsequent analysis (Table S1).

As a comparison, we also downloaded the mRNA expression profile and clinical information of the validation cohort GSE20685 [13] from Gene Expression Omnibus (GEO, https://www.ncbi.nlm.nih.gov/geo/, accessed on 18 March 2021).

### 2.2. Identification of the Differentially Spliced IGAS Events

To identify differences in AS events between primary breast cancer and corresponding normal samples, we applied Student’s *t*-test to identify differentially spliced AS (DSAS) events (*p*-values < 0.05). We divided the patients into high and low immune groups according to the median immune score. Similarly, differentially expressed immunogenes (DEIGs) were identified between high and low immune groups (edgeR R package, *p*-values < 0.05). Furthermore, we defined the gene intersection of DSAS parent genes and DEIGs as IGAS parent genes, followed by the extraction of differentially spliced IGAS events based on IGAS parent genes in DSAS events.

### 2.3. Construction and Evaluation of the IGAS Prognostic Model

To calculate the relationships between IGAS events and overall survival in each AS type, we divided patients into a high or low PSI group by the median PSI value of IGAS events and performed the univariate Cox regression analysis. To remove any IGAS event that might not be an independent prognostic signature, we applied LASSO-penalized multivariate regression [14] that appeared >90% out of 500 repetitions to select prognostic-related IGAS events. The linear combination of IGAS PSI multiplied by a corresponding LASSO-penalized multivariate regression coefficient (λ) representing the weight of the correlation was considered to denote an IGAS prognosis risk score. The IGAS prognosis risk score formula was as follows:

$$\mathrm{Risk}\;\mathrm{score}={\mathrm{PSI}\;\mathrm{of}\;\mathrm{IGAS}}_{1}\times \lambda_{1}+{\mathrm{PSI}\;\mathrm{of}\;\mathrm{IGAS}}_{2}\times \lambda_{2}+\cdots +{\;\mathrm{PSI}\;\mathrm{of}\;\mathrm{IGAS}}_{n}\times \lambda_{n}\;$$

IGAS events with *p*-values < 0.05 in the univariate Cox regression among the seven types were chosen as survival-related IGAS events and were used to establish candidate prognostic signatures. Then, independent prognostic-related IGAS events of the AS types were entered into the LASSO-penalized multivariate regression to construct the final IGAS prognostic model. We used the Kaplan-Meier curves to determine whether the prognostic models could distinguish good or poor prognostic outcomes and applied the receiver operating characteristic (ROC) curve to judge the discriminatory ability of each prognostic model at 5-year.

To further validate the clinical value of the IGAS prognostic model, we assessed the prognostic ability of IGAS risk groups and important clinical information using Cox regression analysis. Moreover, we applied the nomogram to predict the probability of survival at 3-year, 5-year, and 7-year in breast cancer patients (rms R package).

### 2.4. Construction of the AS Regulatory Network

Splicing factors (SFs) are crucial regulators of AS events, a limited number of which could adjust and control numerous AS events [15]. Hence, we further explored AS regulatory network of IGAS events and SFs. We firstly obtained a total of 67 experimentally validated SFs from the SpliceAid-F [16] and SpliceAid 2 [17] databases (Table S2), and also identified differentially expressed SFs (DESFs) and survival-related SFs using differential expression analysis as well as survival analysis, respectively (*p*-values < 0.05). Furthermore, the potential relationships between IGAS events and DESFs were established by calculating the Spearman correlation between the PSI values of AS events and the expression levels of SFs. Finally, we constructed the AS regulatory network by extracting IGAS-DESF interaction partners with *p*-values < 0.05 and |correlation coefficients (cor)| > 0.4.

### 2.5. Prediction of Immunotherapeutic and Chemotherapeutic Response

Following the approval of immune checkpoint inhibitors as common drugs for breast cancer, immunotherapy has emerged as a promising approach for cancer treatment [18], particularly immune checkpoint blockade therapy with immune checkpoints CTLA-4 and PD-1 [19]. Therefore, we assessed the difference in immunotherapeutic responses between the high and low risk groups to reveal their potential for cancer treatment by immune checkpoint blockade. The Tumor Immune Dysfunction and Exclusion (TIDE, http://tide.dfci.harvard.edu/, accessed on 14 May 2021) [20] is a computational method, which integrates the expression signatures of T cell dysfunction and T cell exclusion to model tumor immune evasion. We performed TIDE to assess the individual likelihood of responding to immunotherapy (Wilcoxon test, *p*-values < 0.05). Then, we also applied a subclass mapping approach [21] to predict the clinical response of the IGAS risk groups to immune checkpoint blockade (CTLA-4 and PD-1) by comparing the expression profile of our defined IGAS risk groups with another published dataset containing 47 melanoma patients who responded to immunotherapy [22].

Chemotherapy is one of the basic treatments for breast cancer patients. The Genomics of Drug Sensitivity in Cancer (GDSC) database [23] (www.cancerRxgene.org, accessed on 14 May 2021) is a public resource for drug sensitivity in cancer cells and molecular markers of drug response. Five commonly used drugs were selected: camptothecin [24], docetaxel [25], methotrexate [26], paclitaxel [27], and vinblastine [28]. The pRRophetic algorithm [29] based on the GDSC database was performed to estimate drug response. We assessed the samples’ half-maximal inhibitory concentration (IC50) in the IGAS risk groups using ridge regression to predict drug response and used 10-fold cross-validation based on the GDSC training set to assess prediction accuracy (Wilcoxon test, *p*-values < 0.05).

### 2.6. Identification of the IGAS Clusters

To obtain a steady classification, we applied an unsupervised clustering algorithm (ConsensusClusterPlus R package). The IGAS clusters were determined using hierarchical clustering (k-means clustering algorithm and Euclidean distance) on the prognostic-related IGAS events. Survival analysis was performed to determine the associations between IGAS clusters and overall survival. The different PSI values of the IGAS prognostic signatures and immune and stromal scores in each IGAS cluster were assessed using the Kruskal-Wallis test (*p*-values < 0.05).

### 2.7. Correlation of Immune Cell Infiltration between IGAS Prognosis Signatures and Clusters

On account of tumor-infiltrating immune cells playing essential roles in cancer development and progression, the infiltration levels of immune cell populations were quantified by ssGSEA and systematically correlated with IGAS prognosis signatures and clusters. Firstly, we selected the genesets from Bindea et al. [30], including 461 genes, for predicting the abundance of 24 immune cells. Secondly, we compared the infiltration levels of immune cells by the Wilcoxon test and identified the difference between high and low risk groups (*p*-values < 0.05). Next, we performed correlation analysis to identify the correlation between IGAS prognostic signatures, immune cell infiltration level and its marker genes. In addition, we assessed the differences in immune cell infiltration of each IGAS cluster (Kruskal-Wallis test, *p*-values < 0.05). Considering the vital role of the TME in prognosis, we applied multivariate Cox regression analysis to explore the prognostic significance of immune cells in each IGAS cluster.

## 3. Results

### 3.1. The Differentially Spliced IGAS Event Is an Important Part of AS

**Figure 1:**
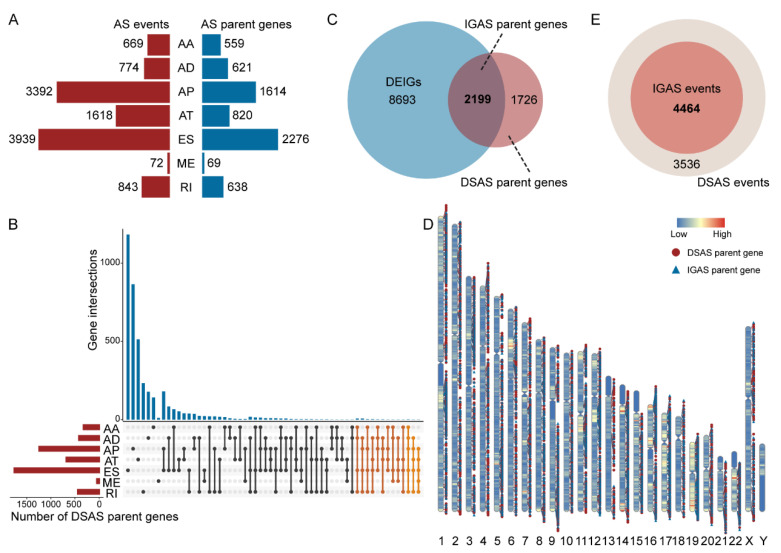
Overview of IGAS events and parent genes in breast cancer. (**A**) The number of overall AS events and parent genes for each AS type. (**B**) UpSet plot of interactions among the seven AS types of detected DSAS parent genes. (**C**) Venn plot of the intersection of DSAS parent genes and DEIGs. (**D**) The location distribution of DSAS and IGAS parent genes on the chromosomes, respectively. (**E**) Venn plot of the intersection of DSAS and IGAS events.

Overall, we identified a total of 11,307 AS events of 4908 genes in breast cancer patients, and ES was the most common AS type (>33%, Figure 1A). Differential AS analysis showed up to 8000 DSAS events of 3925 genes. ES was also the most common type among all dysregulated AS events, followed by AP, AT, RI, AD, AA, and ME (Table S3). In particular, Figure 1B demonstrated that 4–5 types of AS patterns can occur in specific parent genes of DSAS (two orange sections), such as RASSF7, MOK, IL32, and NR1H3. We identified the DEIGs using differential expression analysis. Interestingly, we observed that more than half of DSAS parent genes belonged to DEIGs, called IGAS parent genes (Figure 1C). As previously reported, pre-mRNAs of human immune-related genes are known to undergo extensive AS [31]. Furthermore, we investigated the distribution of DSAS and IGAS parent genes on chromosomes and detected that they were predominant on Chr1, Chr2, Chr17, and Chr19 and did not appear on ChrY (Figure 1D and Table S4). Eventually, we extracted 4464 differentially spliced IGAS events among DSAS events based on IGAS parent genes as the subject of the subsequent analysis (Figure 1E and Table S5).

In addition, we calculated IGAS events for ER+ vs. ER− subgroups, PR+ vs. PR− subgroups, HER2+ vs. HER2− subgroups, and triple-negative breast cancer (TNBC) vs. non-TNBC subgroups, respectively (Figure S1A). We identified 6492, 6115, 2663, and 6339 IGAS events in ER, PR, HER2, and TNBC subgroups, respectively. By comparing the differentially spliced IGAS events of each subgroup, we found multiple intersection patterns among subgroups (Figure S1B). There were 604 common IGAS that existed in all subgroups. Moreover, we detected specific IGAS events in each subgroup, with cancer vs. normal subgroups having the most specific IGAS events, indicating the importance of each pathological subtype.

### 3.2. The IGAS Prognostic Model Is an Independent Prognostic Factor

To investigate the relationship between IGAS events and OS of patients, we performed a univariate Cox regression analysis of the 4464 differentially spliced IGAS events. The result showed up to 489 survival-related IGAS events occurring in 354 genes (Table S6). Among all survival-related IGAS events, AP, ES, and AT were the TOP3 most common type (approaching 85%), followed by AD, RI, AA, ME (Table S3). The circle plot showed the distribution of survival-related IGAS parent genes in chromosomes (Figure S2A). Most survival-related IGAS events occurred with 1-2 AS types and could serve as protective factors (HR < 1). For each AS type, the hazard ratios (HRs) and 95% confidence intervals (95% CIs) of the TOP10 IGAS events with the smallest *p*-values are visualized in Figure S2B–H. By further analyzing biological functions of survival-related IGAS events, we found that they were significantly enriched in several biological processes (Figure S2I) and KEGG pathways (Figure S2J). For example, the MAPK signaling pathway was crucial for apoptosis and tumor induction.

In each AS type using LASSO-penalized multivariate regression, prognostic models exhibited significant power to distinguish well from poor outcomes (*p*-values < 0.05, Figure S3A–G). Especially, the prognostic model based on the single AT model displayed the greatest discriminatory ability, with an AUC of 0.856, and the single ES or AP prognostic model also performed promising discriminatory ability, with an AUC > 0.75 (Figure S3H).

**Figure 2:**
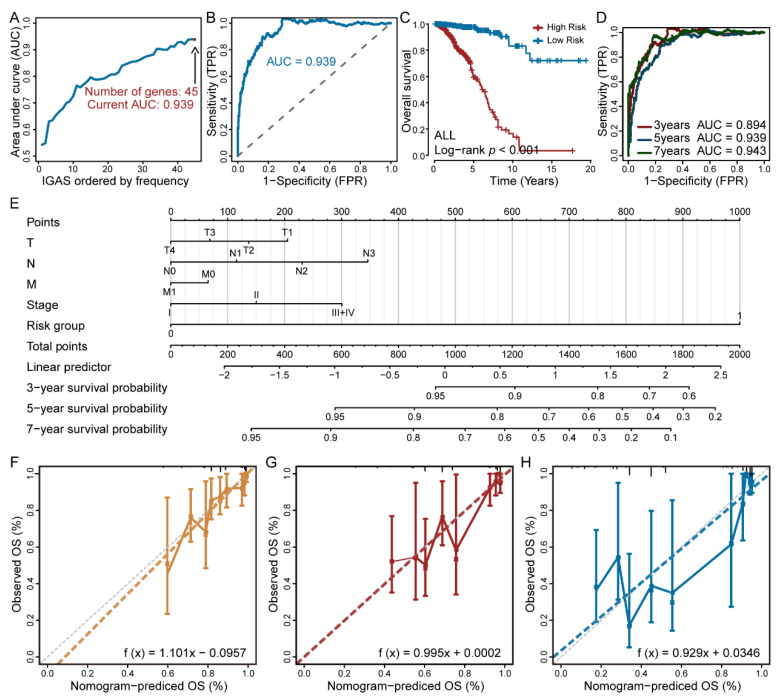
Construction and validation of the IGAS prognostic model in predicting overall survival of breast cancer. (**A**) During the construction of the final IGAS prognostic model, the AUC values were calculated when selecting different prognosis-related IGAS events. (**B**) Maximum AUC value in ROC curves and the number of optimal prognosis-related IGAS events in the final IGAS prognostic model. (**C**) Kaplan-Meier curve of the final IGAS prognostic model. (**D**) The ROC curves with AUCs at 3-year, 5-year, and 7-year of the final IGAS prognostic model, respectively. (**E**) IGAS-clinic nomogram for the IGAS risk groups and clinicopathological characteristics predicting 3-year, 5-year, and 7-year overall survival. (**F**–**H**) The calibration curves for the 3-year, 5-year, and 7-year overall survival predictions, respectively.

To obtain the final IGAS prognostic model, we selected all independent prognostic-related IGAS events of each AS type, and further assessed them as candidates by the LASSO-penalized multivariate regression. The result suggested that the final prognostic model of all AS types exhibited significant power to distinguish good or poor outcomes in breast cancer (*p*-values < 0.001, AUC = 0.939, Figure 2A–C). The ROC curves confirmed that the final IGAS prognostic model had the highest efficiency than other prognostic models of specific AS types (Figure 2D and Table S7). We also performed 1000 random samples to validate the final IGAS prognostic model (80 percent of the samples were randomly selected), and the results showed good stability (AUC > 0.89, Figure S4A). Notably, multiple prognosis-related IGAS events occurring in DAPL1, MAATS1, and AKR1C2 induced good survival (Figure S4B). The above results strongly suggested that IGAS events had not only important biological functioning, but also potential clinical value.

Independent predictive value of the IGAS prognostic model for breast cancer patients with complete clinicopathological characteristics was assessed using Cox regression analysis. Univariate Cox regression analysis indicated certain prognostic values of the IGAS prognostic model, N stage, M stage, pathological stage, and the number of positive lymph nodes in OS and RFS (Table S8). The results of multivariate Cox regression analysis indicated that the IGAS prognostic model was still a robust independent prognostic factor, and additionally, ER status showed independent prognostic value (Table S8). Given the prognostic importance of ER status in breast cancer, we further analyzed the differences in survival of patients with different ER statuses based on the high and low risk groups of the IGAS prognostic model, respectively. Kaplan-Meier analysis revealed that there was still a significant survival difference between the high and low risk groups when stratifying for ER+ and ER− subgroups of breast cancer patients separately (Figure S4C,D). We also found that the probability predicted by the IGAS-clinic nomogram showed optimal agreement with the ideal reference lines for OS prediction (C-index = 0.805, Figure 2E–H).

### 3.3. Diverse Regulatory Patterns of IGAS Events in the AS Regulatory Network

**Figure 3:**
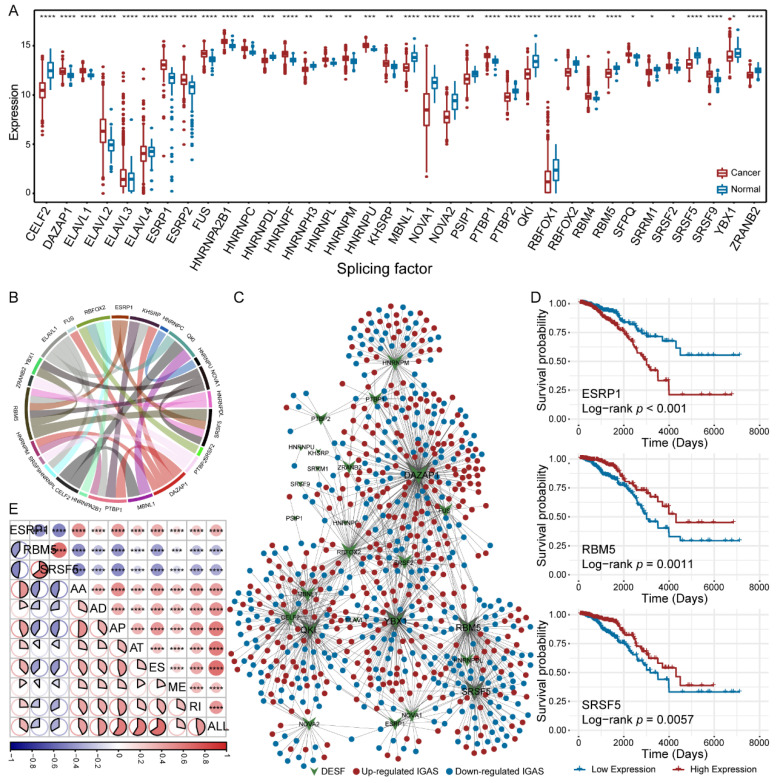
AS regulatory network. (**A**) The difference of expression of the DESFs between primary breast cancer and corresponding adjacent normal tissues. The asterisk character represents the significance of the expression discrepancy. (**B**) The co-expression relationships between DESFs. (**C**) AS regulatory network of IGAS events and DESFs. Ellipse nodes indicate IGAS events that were up-regulated (red dots) or down-regulated (blue dots), and V nodes indicate SFs that were differentially expressed. (**D**) Kaplan-Meier curves of survival-related SFs (ESRP1, RBM5, and SRSF5), respectively. (**E**) The correlation between the risk scores of prognostic models and the expression of survival-related SFs. The size and color of the circle or pie represent the weight and tendency of the correlation coefficient, respectively. The asterisk character represents the significance of the difference or correlation coefficient, * *p* < 0.05; ** *p* < 0.01; *** *p* < 0.001; **** *p* < 0.0001.

Given the important regulatory role of SF on AS events, we established the AS regulatory network to explore the association between DESFs and differentially spliced IGAS events. Firstly, we identified 36 DESFs with aberrant expression in breast cancer (Figure 3A). Then, we recognized co-expression relationships among DESFs using Spearman correlation analysis and found that RBM5 was significantly associated with six DESFs (|cor| > 0.4, Figure 3B). Furthermore, we constructed the AS regulatory network by calculating the correlation between the PSI values of IGAS events and the expression levels of DESFs. Figure 3C has illustrated the AS regulatory network, which contains 721 nodes (696 IGAS events and 25 DESFs) and 1183 edges. By analyzing the topological properties of the network, we sought five hub DESFs (DAZAP1, YBX1, RBM5, QKI, and SRSF5) with TOP5 degree. Especially, DAZAP1 was significantly associated with nearly 1/3 of IGAS events, which was crucial to the network. Interestingly, one IGAS event could be regulated by several SFs, which reflected a complex cooperative or competitive relationship between SFs and explained the diversity of AS events generated by a few SFs.

We also identified three survival-related SFs, including ESRP1, RBM5, and SRSF5 (Figure 3D). Among them, RBM5 and SRSF5, the hub nodes of the AS regulatory network, whose low expression predicted poor prognosis. KEGG pathway enrichment analysis showed that three SFs associated IGAS genes have specific biological functions, respectively (Table S9). For example, EGFR-associated IGAS genes were significantly enriched in multiple signaling pathways, such as PI3K-Akt signaling pathway, VEGF signaling pathway, and p53 signaling pathway, while RBM5- and SRSF5-associated IGAS genes were significantly enriched in primary immunodeficiency and biosynthesis of cofactors, respectively. Interestingly, all survival-related SFs are powerfully associated with each prognostic model (Figure 3E). Among three survival-related SFs, ESRP1 had positive correlations, and RBM5 and SRSF5 had negative correlations with all prognostic models, consistent with survival analysis.

### 3.4. IGAS Prognosis Signatures and Immune Cells Demonstrated Consistent Trends of Correlation

**Figure 4:**
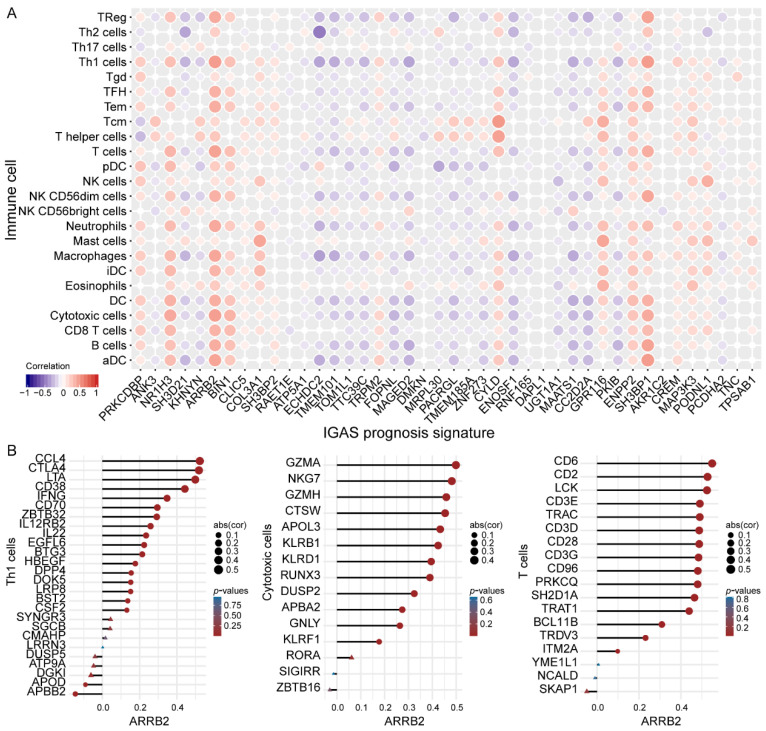
The correlation of IGAS prognosis signatures with immune cells and their marker genes. (**A**) The correlation of IGAS prognosis signatures with each immune cell, respectively (range from blue to red, which represent significant negative correlation and significant positive correlation, non-significant correlation part was not displayed yet). (**B**) The illustration showed the correlation between ARRB2 and the marker genes of Th1, cytotoxic and T cells, respectively. The circle node represents the significant correlation, and the triangle node represents the non-significant correlation yet. The size of a node represents the weight of the correlation coefficient.

We further characterized the association of immune cells with the IGAS prognostic model, particularly the difference in the infiltration levels of immune cells among risk groups and the correlation with IGAS prognosis signatures. The violin plot visualized the abundance of 24 infiltrating immune cell types in the high and low risk groups (Figure S5A). Notably, more than half of the immune cells differed significantly between IGAS risk groups, most of which had higher infiltration levels in the low-risk group (such as B cells, CD8 T cells, NK cells, etc.). Moreover, we observed that some IGAS prognosis signatures were significantly associated with immune cells and consistent correlation tendency (Figure 4A and Table S10). Interestingly, we also found that T lymphocytes, especially Th1 cells, cytotoxic cells, and T cells, possessed significant correlations with a range of IGAS prognosis signatures (Table S11). In particular, the correlation coefficients of ARRB2 with Th1 cells, cytotoxic cells, and T cells were all higher than 0.45 (Figure S5B). Furthermore, there were also statistically significant correlations between ARRB2 and most marker genes of Th1 cells, cytotoxic cells, and T cells, respectively (Figure 4B). In the validation cohort GSE20685, we observed a significant and consensus correlation between immune cells and most IGAS prognosis signatures, consistent with the TCGA cohort (Figure S5C). Previous studies have shown that tumor-infiltrating lymphocytes (TILs) are important prognostic indicators [32], as well as increased levels of immune infiltration lead to a reduced risk of death and distant recurrence [33]. To a certain extent, both the IGAS prognosis signatures and the infiltration levels of immune cells (particularly TILs) explain the significant difference in overall survival among the IGAS risk groups.

### 3.5. Differences in Sensitivity of Immunotherapy and Chemotherapy in the IGAS Risk Groups

**Figure 5:**
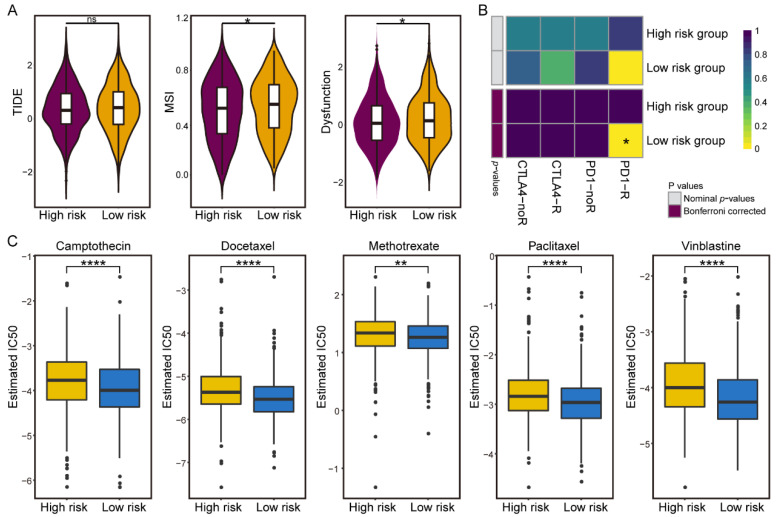
Differential putative immunotherapeutic and chemotherapeutic response in IGAS risk groups. (**A**) TIDE, MSI, and T cell dysfunction scores in high and low risk groups. (**B**) The Submap analysis manifested that low-risk group patients could be more sensitive to PD-1 immunotherapy. (**C**) The boxplots illustrated differential chemotherapeutic response based on IC50 for five drugs between the high and low risk groups. The asterisk character represents the significance of the difference, * *p* < 0.05; ** *p* < 0.01; **** *p* < 0.0001; ns: not significant.

To investigate the worth of the IGAS prognostic model in clinical therapies, we explored the possibilities of immunotherapy and chemotherapy in different risk groups. We evaluated the potential clinical efficacy of immunotherapy in different IGAS risk groups using TIDE (Figure 5A). Although TIDE scores were not significantly different in the IGAS risk groups, we observed a higher microsatellite instability (MSI) in the low-risk group, which remains consistent with the results of previous studies that a higher MSI is associated with a better prognosis and is sensitive to checkpoint immunotherapy [34]. Besides, T-cell dysfunction scores were higher in the low-risk group than in the high-risk group. We further applied the Submap algorithm to predict the therapeutic effect of immune checkpoint blockades (CTLA-4 and PD-1). The result demonstrated that patients in the low-risk group with higher levels of immune infiltration had higher sensitivity to anti-PD-1 immunotherapy (Bonferroni correction *p*-values = 0.024) (Figure 5B). It has been shown that TILs are still useful as immune checkpoint inhibitors [35], and a larger number of TILs is associated with higher patient response rates [36]. The presence of immune cells controlled by PD-1 and PD-L1 in TILs may be a prerequisite for the usefulness of immune checkpoint inhibitors [37].

Considering that chemotherapy is a common treatment for breast cancer, we attempted to assess the response of representative chemotherapeutic drugs in IGAS risk group patients. Remarkably, all five chemotherapeutic drugs could be observed to present a significant response sensitivity to the low-risk group compared with the high-risk group (Figure 5C). Studies have demonstrated that incremental increases in the level of TILs both intra- and peri-tumor predict an improved response to chemotherapy and survival in patients [38]. It is worth noting that the low-risk group with high levels of immune infiltration in our study showed a trend consistent with previous studies. The differential sensitivity of IGAS risk groups to immunotherapy and chemotherapy is expected to benefit from clinical therapeutics and further guide the personalized medicine of breast cancer patients.

### 3.6. Prognosis-Related IGAS Clusters Demonstrated the Heterogeneity in Breast Cancer

**Figure 6:**
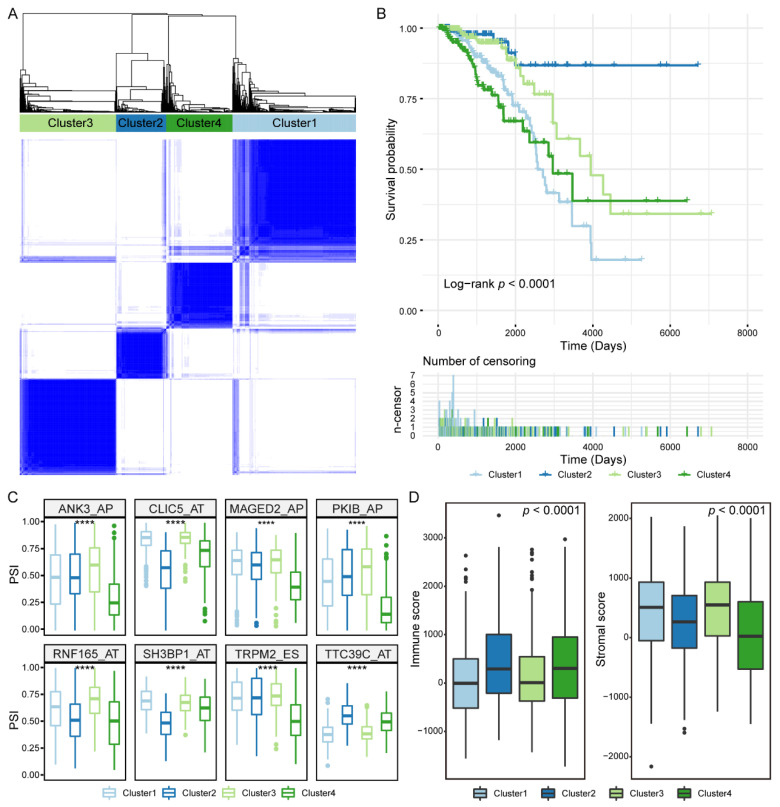
Unsupervised classification identified four IGAS clusters. (**A**) The consensus clustering matrix showed the optimal four IGAS clusters. (**B**) Kaplan-Meier curves of patients within different IGAS clusters on overall survival. (**C**) The difference of PSI values of partial IGAS prognosis signatures in each IGAS cluster. The asterisk character represents the significance of the difference, **** *p* < 0.0001. (**D**) Immune characteristics, including immune and stromal scores, exhibited significant differences in IGAS clusters.

Our findings suggested that prognosis-related IGAS events vary widely at an individual level. To discriminate the distinct patterns of AS, we clustered prognosis-related IGAS events using a consensus unsupervised clustering approach. The consensus matrix of four IGAS clusters with robust classification was defined (Figure 6A). The number of four IGAS clusters was determined as follows: Cluster1 (*n* = 337, 36.7%), Cluster2 (*n* = 137, 14.9%), Cluster3 (*n* = 263, 28.7%), Cluster4 (*n* = 181, 19.7%). To assess the difference of IGAS clusters in overall survival, we applied Kaplan-Meier analysis to ascertain the survivability of distinct IGAS clusters. As shown in Figure 6B, the IGAS clusters had significantly different prognosis with important heterogeneity, in which both Cluster1 and Cluster4 were associated with poor prognosis of patients, while Cluster2 was associated with good prognosis of patients. Furthermore, we found that IGAS clusters had different IGAS prognosis signatures (Figure 6C) and were significantly enriched in different biological processes (*p*-values < 0.01, Table S12). In particular, Cluster1 and Cluster4 were significantly associated with important processes in cancer development and progression, such as protein polyubiquitination and negative regulation of potassium ion transport. By further comparing immune characteristics between IGAS clusters, we found that Cluster2 and Cluster4 had a higher immune score, while Cluster1 and Cluster3 had a higher stromal score (Figure 6D). It suggested that specific immune cell-type mixtures, rather than the total number of immune cells in the complex TME, may be responsible for the differential prognostic ability of the IGAS clusters.

### 3.7. Immune Cells Exhibited Different Prognostic Abilities in Different IGAS Clusters

**Figure 7:**
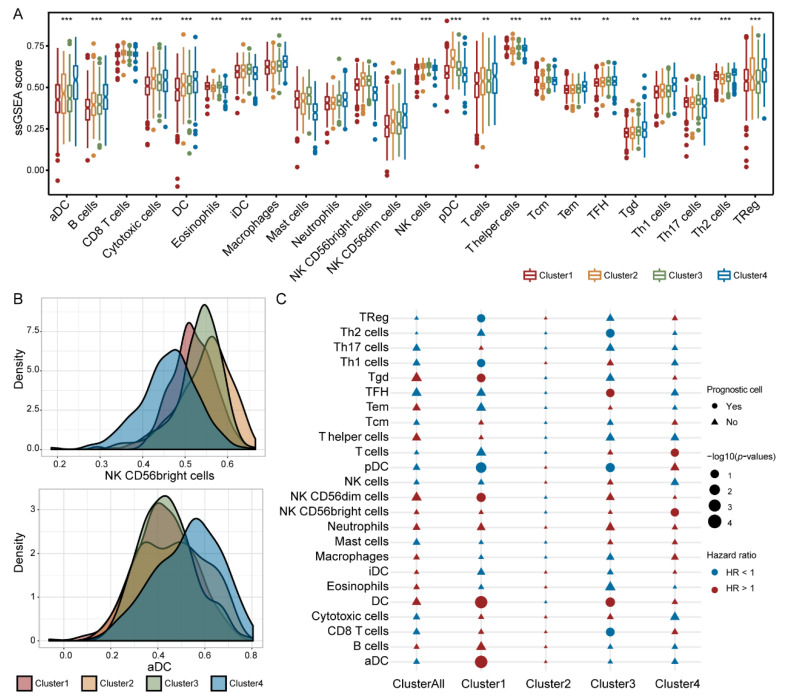
Prognostic significance of immune cells in each IGAS cluster. (**A**) The infiltration levels of immune cells among IGAS clusters. The red, yellow, blue, and green boxplots represent each IGAS cluster, respectively. The asterisk character represents the significance of the difference, ** *p* < 0.01; *** *p* < 0.001. (**B**) The density plot of NK CD56bringht cells and activated dendritic cells of immune cell infiltration distributions among four IGAS clusters, respectively. (**C**) Estimation of the prognostic significance of each immune cell for overall survival in the whole cohort and each IGAS cluster. The circle represents the prognosis-related cell, the triangle node represents the non-prognosis-related cell yet. The size and color of the circle represent –log10 (*p*-values) and hazard ratio (blue circle was HR < 1, red circle was HR > 1), respectively.

Considering the vital role of immune cells, we evaluated their infiltrating differences and prognostic capacity in the IGAS clusters. In the IGAS clusters, each immune cell type expressed extremely significant differences (Figure 7A,B). We likewise investigated whether each immune cell of the IGAS cluster plays an important role in prognosis using the multivariate Cox regression analysis. In the whole cohort, neither one of the immune cells could predict a better prognosis. However, a within-clusters analysis indicated that the prognostic significance of immune cells in the three IGAS clusters was significant, diverse, and even opposite (Figure 7C). One or more immune cells could serve as prognosis-related cells in each IGAS cluster, besides Cluster2. For example, Treg, Th1 cells, Tgd, DC, pDC, aDC, and NK CD56dim cells of immune cells could be prognosis-related cells in Cluster1, Th2 cells, TFH, DC, pDC, and CD8 T cells of immune cells could be prognosis-related cells in Cluster3, as well as T cells, and NK CD56bringht cells could be prognosis-related cells in Cluster4. In particular, most prognosis-related immune cells had prognostic significance in only one IGAS cluster. These results indicated that immune cells had the ability to act as prognostic indicators in IGAS clusters.

## 4. Discussion

AS not only regulates gene expression levels, but also ensures gene product diversity. In mammals, AS, which occurs in more than 90% of genes, is particularly prevalent in the immune system [39]. There is growing evidence that AS has a tremendous impact on various tumorigenic processes [40], such as cancer onset, progression, angiogenesis, and immune escape. Although many immunogenes, such as pro-inflammatory cytokines and chemokines [41], have been found to undergo AS, comprehensive profiling at the whole genome level could better characterize IGAS as potential prognostic and predictive signatures in breast cancer patients is still lacking. In our study, we obtained RNA splicing data from the TCGA SpliceSeq database, extracted IGAS events by filtering immunogenes, and performed a systematic profiling analysis to elucidate its important roles in breast cancer, which included prognostic ability, association of clinical information, sensitivity to immuno/chemotherapy, regulation of the AS network, identification of heterogeneous subtypes, and differences in the infiltration level of immune cells.

We specifically identified the differentially spliced IGAS events, screened and obtained the prognosis-related IGAS events, and constructed prognostic models. In differentially spliced IGAS events and survival-related IGAS events, AP, AT, and ES were the major AS types, consistent with other studies [42]. Notably, multiple isoforms of TNC were generated mainly through the ES type of AS and were significantly associated with survival. Evidence showed that TNC affects the invasion and growth of cell lines in vitro and is an important marker in breast malignancy [43]. Tumorigenesis is a complex regulatory network, therefore, the IGAS prognostic model could improve prognostic efficiency by integrating multiple signatures rather than single clinical indicators.

We constructed a potential AS regulatory network between differentially expressed SFs and IGAS events. The overview of the network revealed apparent trends that one IGAS event could be regulated by multiple SFs, which also reflected multiple interactions between IGAS events and SFs. Meanwhile, three survival-related SFs were identified by survival analysis, in which RBM5 and SRSF5 were confirmed to be hub nodes in the AS regulatory network. There is evidence that overexpression of RBM5 is involved in the regulation of AS and suppresses tumor growth by controlling apoptosis and cell cycle [44]. In particular, there was an extremely robust correlation between survival-related SF and IGAS prognostic models, a phenomenon that may re-emphasize the complex cooperative or competitive relationship between SFs and IGAS events [45].

Immunotherapy has shown promising efficacy in the clinical treatment of various cancers. The TIDE algorithm could effectively predict immune responses in melanoma and non-small cell lung cancer by characterizing dysfunctional T cells and cytotoxic T lymphocytes [20]. We extended the TIDE algorithm to breast cancer patients in an attempt to assess differences in immunotherapy response across IGAS risk groups. The low-risk group had higher T-cell dysfunction scores, indicating that their patients are likely to benefit from immunotherapy. We used the Submap algorithm to predict the response to anti-PD1 and anti-CTLA4 immunotherapy in different risk groups and indicated that the low-risk groups might respond better to anti-PD-1 immunotherapy. These results further suggested that the IGAS risk groups might provide new insights for exploring breast cancer therapeutics in the future.

Breast cancer is not only tumor cells, but also influenced by the TME. The TME is now recognized as a key factor in tumorigenesis and progression, serving as a prognostic factor or a potential therapeutic target [46]. In our study, there was higher immune infiltration of TILs in the low-risk group, consistent with previous studies [33]. Many reports have shown that the number and phenotype of TILs determine the clinical outcome [47]. T lymphocytes are the predominant tumor lymphocyte type in the TME. In breast cancer, as in many other cancer types, tumors rich in CD8 T cells, cytotoxic cells, and TFH were associated with a better prognosis. Similarly, dendritic cells and natural killer cells followed a similar trend. In contrast, the different prognostic capabilities exhibited by immune cells in IGAS clusters may be related to the combined effect of other clinical information.

## 5. Conclusions

In summary, our study provided a systematic analysis of IGAS events in breast cancer to assess the association between prognosis signatures, AS regulatory network, infiltration level of immune cells, sensitivity to immuno/chemotherapy, and heterogeneous IGAS clusters. This comprehensive analysis remarkably enhanced our understanding of IGAS events and TME, which may be most valuable in deciphering the underlying mechanisms of IGAS in oncogenesis and provided clues to molecular diagnostic biomarkers and therapeutic targets for further validation.

## Data Availability

In our study, the mRNA AS patterns of breast cancer were obtained from TCGA SpliceSeq (https://bioinformatics.mdanderson.org/TCGASpliceSeq/, accessed on 26 August 2020). The gene expression RNA-Seq data and corresponding clinicopathological characteristics of breast cancer were obtained from TCGA (https://tcga-data.nci.nih.gov/tcga/, accessed on 26 August 2020). Immune and stromal scores of breast cancer samples were downloaded from ESTIMATE (https://bioinformatics.mdanderson.org/estimate/, accessed on 26 August 2020). Validation cohort GSE20685 was downloaded from GEO (https://www.ncbi.nlm.nih.gov/geo/, accessed on 18 March 2021).

## Supplementary Materials

The following supporting information can be downloaded at: https://www.mdpi.com/article/10.3390/cancers14030595/s1, Figure S1: Differentially spliced IGAS events in different subgroups of breast cancer, Figure S2: Identification of survival-related IGAS events in breast cancer, Figure S3: Construction of a prognostic model of each AS type in breast cancer, Figure S4: Assessment capabilities of the final IGAS prognostic model, Figure S5: The infiltration levels based on the estimated 24 immune cell types calculated by ssGSEA, Table S1: Clinicopathological characteristics of breast cancer patients in TCGA, Table S2: Information of experimentally validated splicing factors in the SpliceAid-F and SpliceAid 2 databases, Table S3: Statistical information of AS events and parent genes in each phase, Table S4: Statistical information of DSAS and IGAS parent genes on each chromosome, Table S5: Differentially spliced IGAS events and parent genes, Table S6: Survival-related IGAS events and parent genes, Table S7: Statistical information of the ROC curve for each prognostic model, Table S8: The association of IGAS prognostic model and clinicopathological characteristics with OS and RFS, Table S9: Significant enrichment of KEGG pathways of the SF-associated IGAS genesets, Table S10: The correlation of the immune cells with each IGAS prognosis signature, Table S11: Statistical information on the correlation between the immune cells and the IGAS prognosis signatures, Table S12: Significant enrichment of biological processes of each IGAS cluster.
