# Supplementary material for: Identification and Characterization of Immunogene-Related Alternative Splicing Patterns and Tumor Microenvironment Infiltration Patterns in Breast Cancer
Source: Cancers (Basel). 2022 Jan 25;14(3):595. doi: 10.3390/cancers14030595 (PMC8833331; doi:10.3390/cancers14030595)
Supplement: Supplementary file 1 [file cancers-14-00595-s001.zip › Supplementary Figures.pdf]

Supplementary Materials

Identification and Characterization of Immunogene-Relaed Alternative Splicing Patterns and Tumor Microenvironment Infiltration Patterns in Breast Cancer

Shuang Guo, Xinyue Wang, Hanxiao Zhou, Yue Gao, Peng Wang, Hui Zhi, Yue Sun, Yangyang Hao, Jing Gan, Yakun Zhang, Jie Sun, Wen Zheng, Xiaoxi Zhao, Yun Xiao and Shangwei Ning

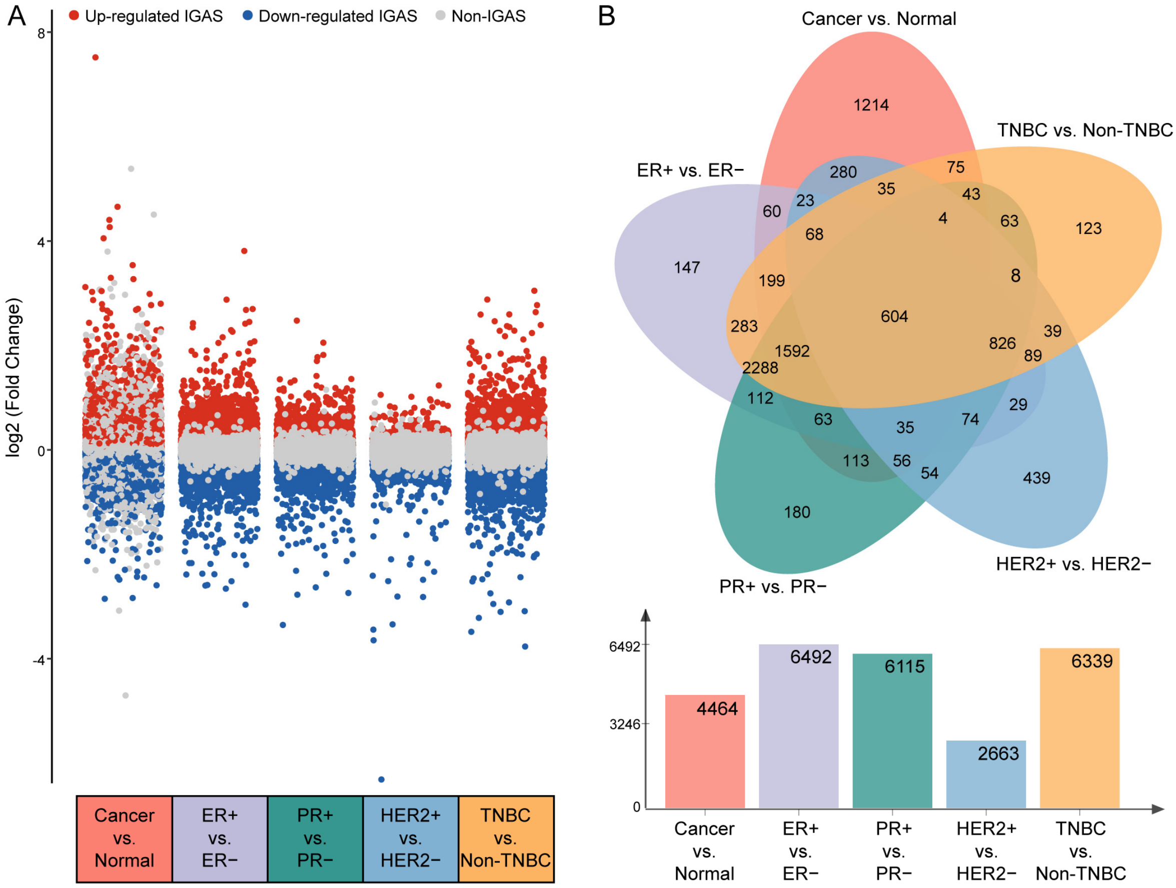

Figure S1. Differentially spliced IGAS events in different subgroups of breast cancer. (**A**) Differentially spliced IGAS events in cancer vs. normal, ER+ vs. ER−, PR+ vs. PR−, HER2+ vs. HER2− and TNBC vs. non-TNBC subgroups, respectively. (**B**) Venn plot of the intersection of IGAS events in different subgroups.

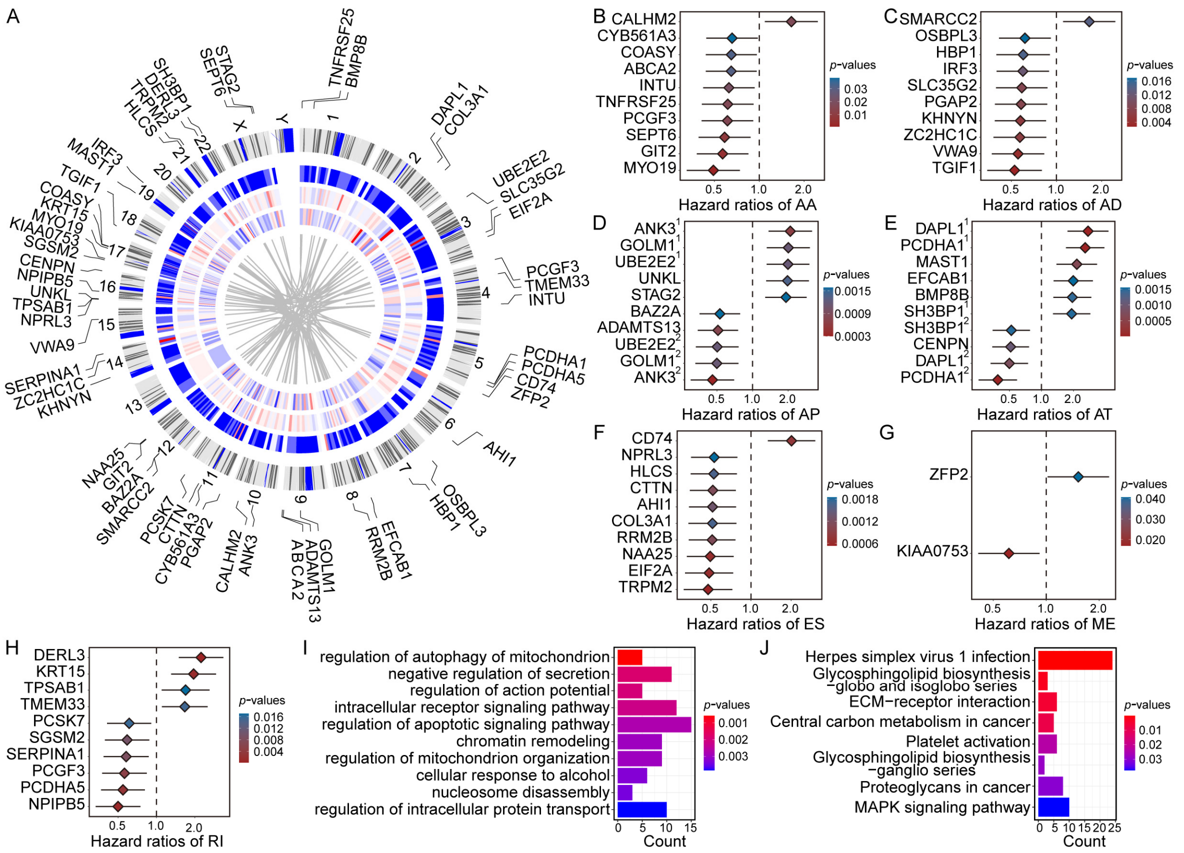

Figure S2. Identification of survival-related IGAS events in breast cancer. (**A**) Circos plots of survival-related IGAS parent genes information in chromosomes. Circos panels from outside to the inside are represented as follows: survival-related IGAS parent genes (TOP10 of each AS type); the genomic axes; chromosomes ideogram; the number of AS type of IGAS parent gene; the log2(fold change) of survival-related IGAS event, which log2(fold change) > 0 / log2(fold change) < 0 was represented with red/blue block; the hazard ratio of survival-related IGAS event, which HR > 1/HR < 1 was represented with red/blue block; and gene interactions. (**B–H**) Forest plots of hazard ratios for survival-related IGAS events in each AS type, respectively (ANK3^{1} is ID:11845-ANK3-AP, ANK3^{2} is ID:11842-ANK3-AP, GOLM1^{1} is ID:86745-GOLM1-AP, GOLM1^{2} is ID:86743-GOLM1-AP, UBE2E2^{1} is ID:63709-UBE2E2-AP, UBE2E2^{2} is ID:63710-UBE2E2-AP, DAPL1^{1} is ID:55686-DAPL1-AT, DAPL1^{2} is ID:55687-DAPL1-AT, PCDHA1^{1} is ID:73765-PCDHA1-AT, PCDHA1^{2} is ID:73766-PCDHA1-AT, SH3BP1^{1} is ID:62132-SH3BP1-AT, SH3BP1^{2} is ID:62134-SH3BP1-AT). (**I,J**) Survival-related IGAS parent genes were significantly enriched for biological processes and KEGG pathways, respectively.

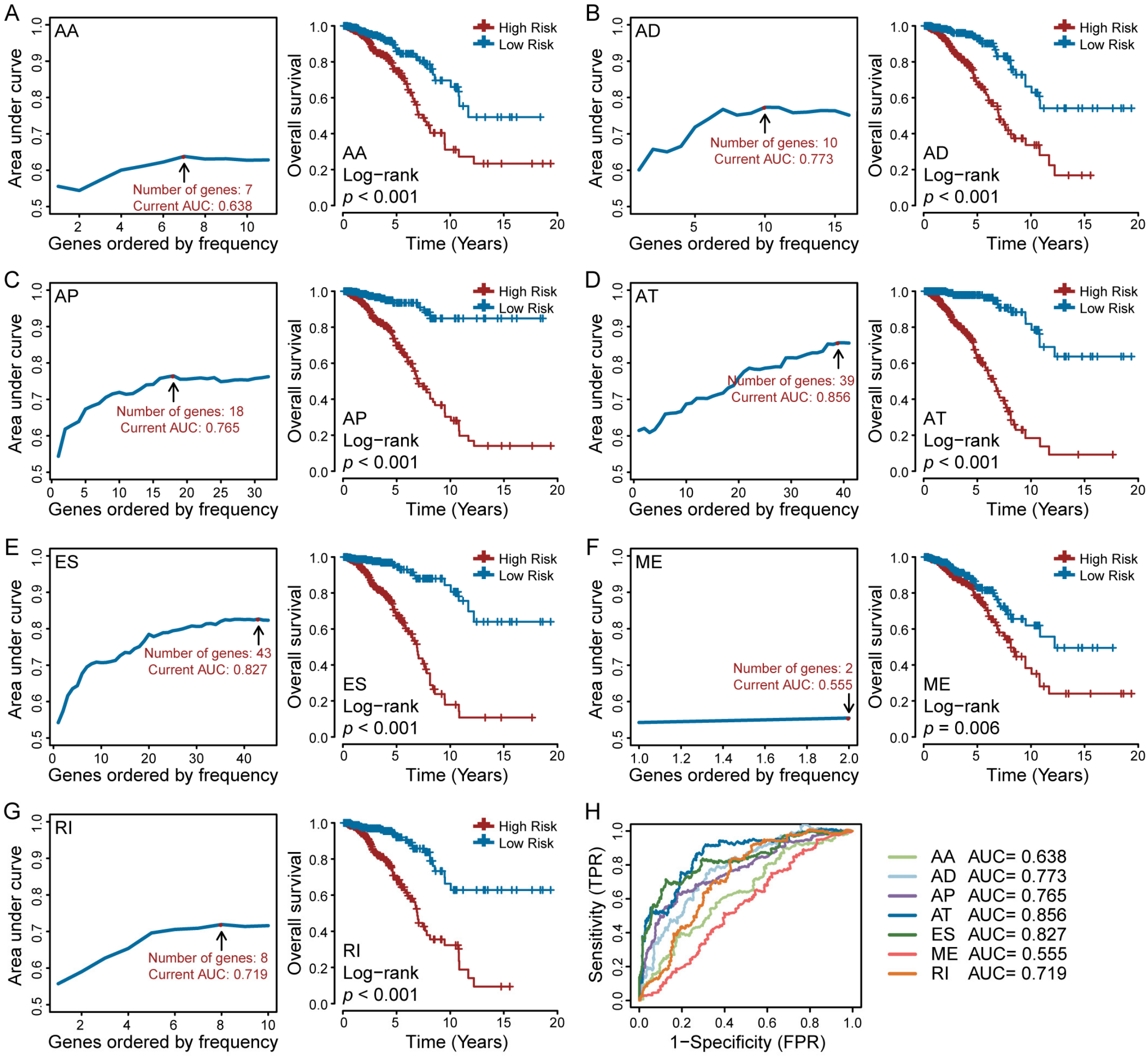

Figure S3. Construction of a prognostic model of each AS type in breast cancer. (**A–G**) The line charts and Kaplan-Meier curves of prognostic models in AA, AD, AP, AT, ES, ME, and RI types, respectively. (**H**) The ROC curves of prognostic models of each AS type at 5-year.

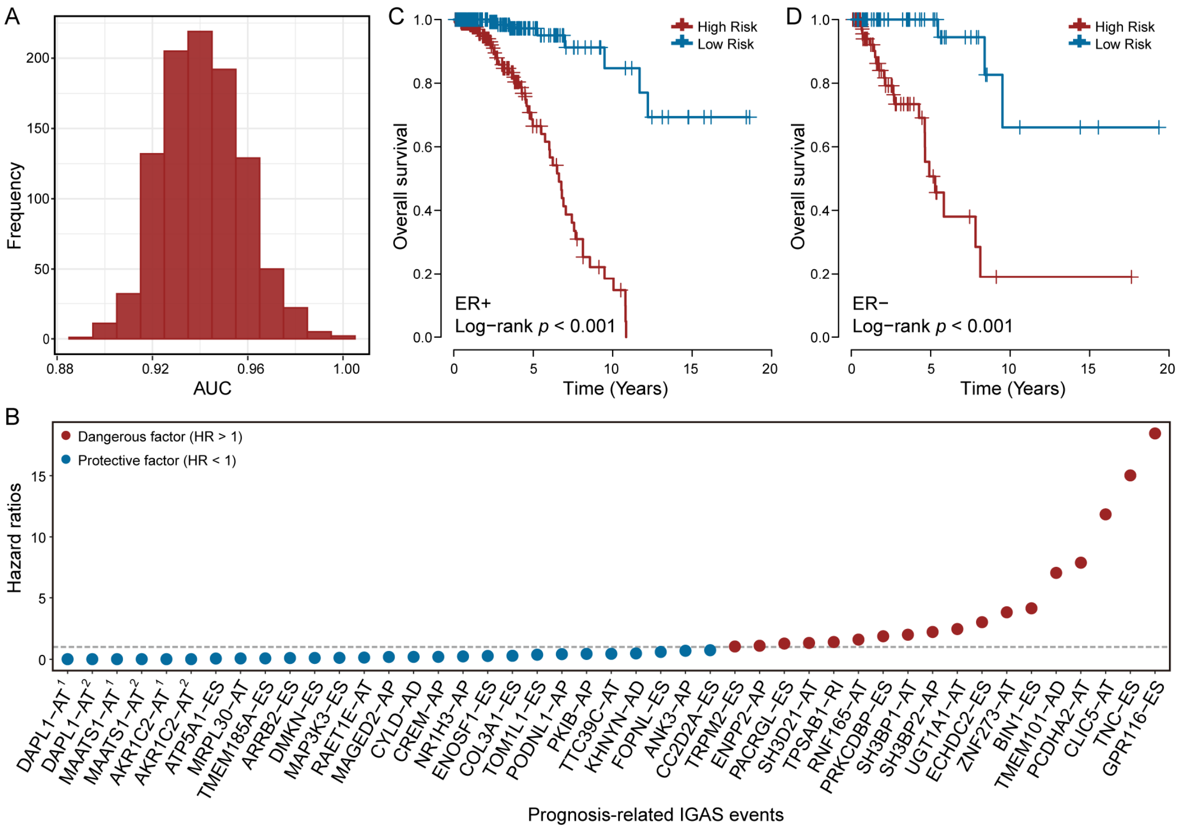

Figure S4. Assessment capabilities of the final IGAS prognostic model. (**A**) The frequency distribution histogram for the AUC value of the IGAS prognostic model in randomly sampling 80% of TCGA breast cancer cohort (1000 repetitions). (**B**) The association of prognosis-associated IGAS events with breast cancer prognosis (DAPL1[1] is ID:55687-DAPL1-AT, DAPL1[2] is ID:55686-DAPL1-AT, MAATS1[1] is ID:66354-MAATS1-AT, MAATS1[2] is ID:66353-MAATS1-AT, AKR1C2[1] is ID:10651-AKR1C2-AT, AKR1C2[2] is ID:10650-AKR1C2-AT). (**C,D**) Kaplan-Meier curves for the final IGAS prognostic model distinguishing prognostic differences for ER+ and ER− populations, respectively.

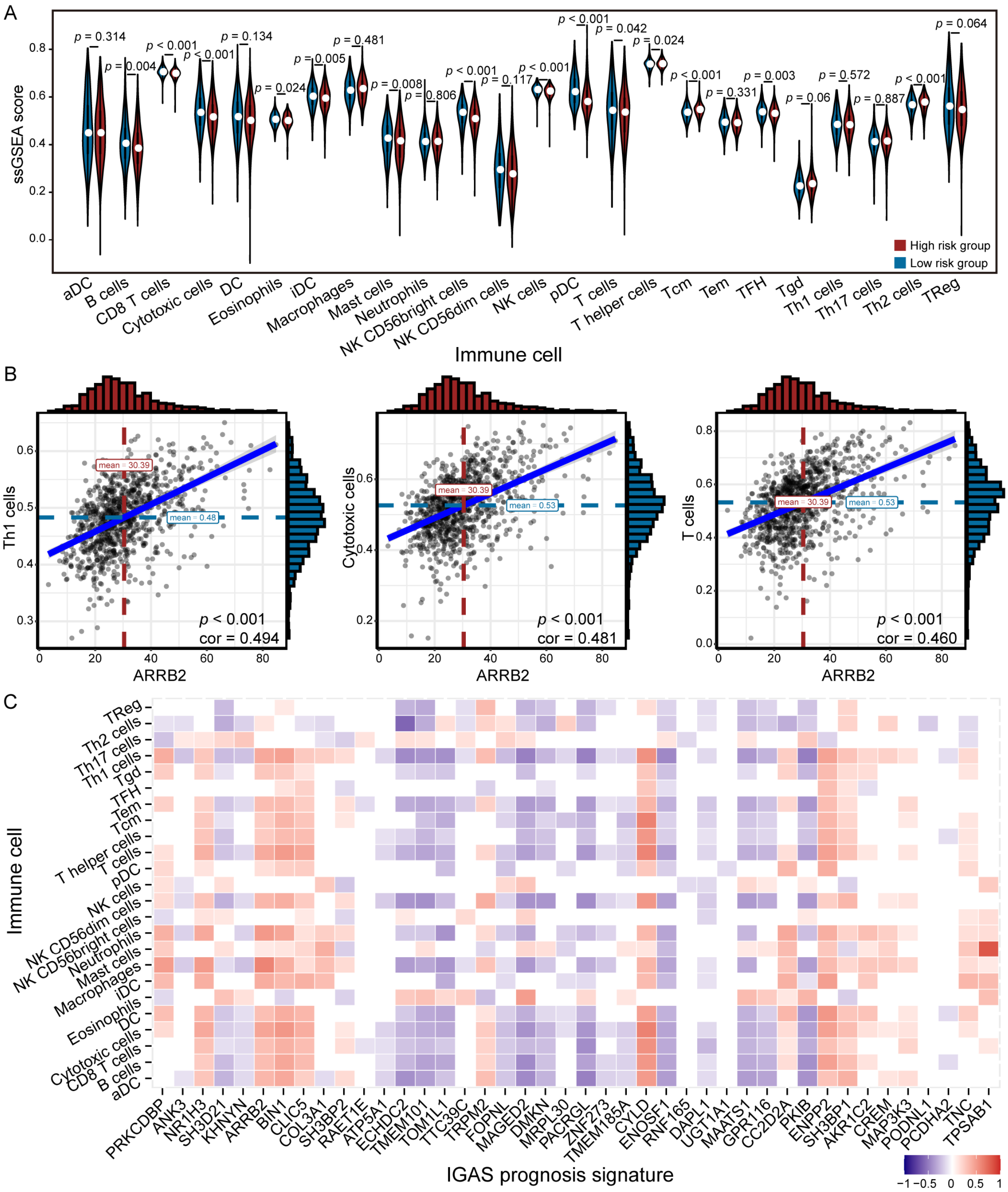

Figure S5. The infiltration levels based on the estimated 24 immune cell types calculated by ssGSEA. (**A**) The distribution of ssGSEA score among the immune cells in the low-risk (blue) and high-risk (red) groups. (**B**) The representative dot plots of correlations among Th1 cells, cytotoxic cells and T cells of immune cells as well as ARRB2 of IGAS prognosis signatures. (**C**) The correlation of IGAS prognosis signatures with each immune cell in validation cohort GSE20685, respectively.
